# Polarized Anti-Inflammatory Mesenchymal Stem Cells Increase Hippocampal Neurogenesis and Improve Cognitive Function in Aged Mice

**DOI:** 10.3390/ijms24054490

**Published:** 2023-02-24

**Authors:** Matanel Tfilin, Nikolai Gobshtis, David Fozailoff, Vadim E. Fraifeld, Gadi Turgeman

**Affiliations:** 1 Department of Molecular Biology, Faculty of Natural Sciences, Ariel University, Ariel 40700, Israel; 2 The Shraga Segal Department of Microbiology, Immunology and Genetics, Faculty of Health Sciences, Center for Multidisciplinary Research on Aging, Ben-Gurion University of the Negev, Beer-Sheva 8410501, Israel; 3 Medical School, Ariel University, Ariel 40700, Israel

**Keywords:** mesenchymal stem cells (MSC), pituitary adenylate cyclase-activating peptide (PACAP), MSC2, systemic inflammation, aging, cognitive decline, hippocampal neurogenesis

## Abstract

Age-related decline in cognitive functions is associated with reduced hippocampal neurogenesis caused by changes in the systemic inflammatory milieu. Mesenchymal stem cells (MSC) are known for their immunomodulatory properties. Accordingly, MSC are a leading candidate for cell therapy and can be applied to alleviate inflammatory diseases as well as aging frailty via systemic delivery. Akin to immune cells, MSC can also polarize into pro-inflammatory MSC (MSC1) and anti-inflammatory MSC (MSC2) following activation of Toll-like receptor 4 (TLR4) and TLR3, respectively. In the present study, we apply pituitary adenylate cyclase-activating peptide (PACAP) to polarize bone-marrow-derived MSC towards an MSC2 phenotype. Indeed, we found that polarized anti-inflammatory MSC were able to reduce the plasma levels of aging related chemokines in aged mice (18-months old) and increased hippocampal neurogenesis following systemic administration. Similarly, aged mice treated with polarized MSC displayed improved cognitive function in the Morris water maze and Y-maze assays compared with vehicle- and naïve-MSC-treated mice. Changes in neurogenesis and Y-maze performance were negatively and significantly correlated with sICAM, CCL2 and CCL12 serum levels. We conclude that polarized PACAP-treated MSC present anti-inflammatory properties that can mitigate age-related changes in the systemic inflammatory milieu and, as a result, ameliorate age related cognitive decline.

## 1. Introduction

Neurogenesis continues throughout life in the dentate gyrus of the hippocampus in many mammals examined thus far and, arguably, also in humans [1,2]. Neurogenesis is associated with spatial learning and memory, as well as other cognitive functions [2]. Consequently, age-related decline in cognitive functions is linked to reduced hippocampal neurogenesis, which, to a great extent, is caused by changes in the systemic inflammatory milieu [3]. Indeed, brain aging is associated with increased systemic levels of anti-neurogenic pro-inflammatory chemokines and cytokines such as CCL11, CCL12, CCL2, CCL19, b2-microglobulin, hepatoglobin and a decreased expression of pro-neurogenic factors such as GDF11 [4]. Thus, ameliorating these pro-inflammatory changes can potentially treat brain aging and related cognitive impairments [5].

Mesenchymal stem cells (MSC) are stromal cells found in a wide range of adult tissues and can be easily isolated and expanded from bone marrow and adipose tissue [6]. MSC are characterized by their ability to differentiate into various mesodermal cell lineages including bone, cartilage and adipose cells [7]. However, much of their therapeutic properties result from their secretome [8]. MSC are known modulators of the immune system and immune responses. MSC were shown to have direct immunosuppressive properties by inhibiting the activation and proliferation of effector T cells (Th1 and Th17) while increasing proliferation of regulatory T (Treg) cells via cell-to-cell contact and the secretion of various soluble factors [9,10]. The abundance of MSC mediators and proposed mechanisms suggests a reciprocal relationship in which MSC may be either immunosuppressors or immune activators [11,12,13]. Akin to immune cells, MSC can also polarize into pro-inflammatory MSC (MSC1) and anti-inflammatory MSC (MSC2) following activation of Toll-like receptor 4 (TLR4) or TLR3, respectively [14,15].

In the present study, we have found that treating MSC with the neuropeptide pituitary adenylate cyclase-activating peptide (PACAP) can polarize MSC towards MSC2-like phenotype. We hypothesized that administration of polarized MSC (pMSC) to aged mice could increase hippocampal neurogenesis and improve cognitive functions. Indeed, we have found that systemic administration of pMSC reduced serum levels of pro-ageing chemokines accompanied by beneficial effects on hippocampal neurogenesis and behavior.

## 2. Results

### 2.1. Culturing MSC in the Presence of PACAP Polarized MSC towards MSC2 with an Anti-Inflammatory Phenotype

**Figure 1:**
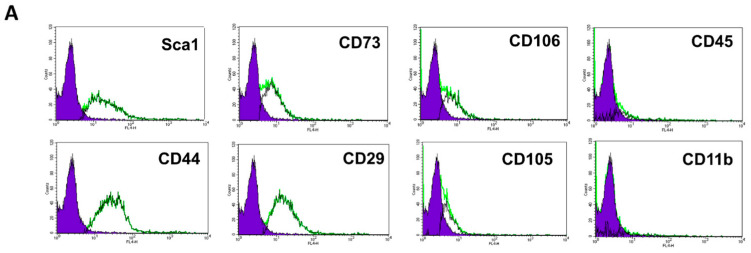

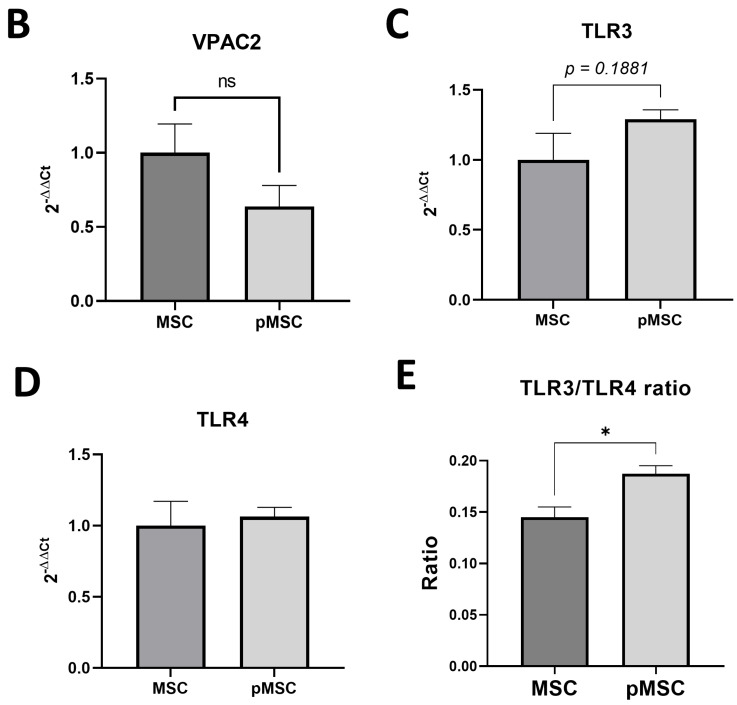
Polarized PACAP-treated MSC maintain mesenchymal phenotype. (**A**). Immunophenotyping of PACAP-treated MSC in flow cytometry presents positive expression of the mesenchymal markers CD106 (>50%), CD29 (>80%), CD44 (>70%), CD73 (>60%) and sca-1 (>40%) but negative expression of the hematopoietic markers CD45 (<8%), and CD11b (<5%). Graphs represent flow cytometry histograms for the expression of the different markers. The negative control histogram is presented with the blue filled histogram. (**B**). Both naïve (N = 4) and PACAP-treated MSC (N = 3) expressed detectable levels of VPAC2 receptor mRNA, as detected in real-time PCR. Since the activation of Toll-like receptor 3 (TLR3) is an established marker of the MSC anti-inflammatory phenotype (MSC2) [15], we administered pituitary adenylate cyclase-activating peptide (PACAP), a neuropeptide with anti-inflammatory properties that is known to upregulate TLR3 and vice versa with TLR4, at 20 nM for 4 days to establish the anti-inflammatory MCS phenotype (MSC2). PACAP treatment of MSC in vitro (pMSC, N = 5) did not increase significantly the expression of TLR3 (**C**) or TLR4 (**D**) but increased the TLR3/TLR4 gene expression ratio (**E**) compared with naïve MSC (N = 4), as detected in real-time PCR. All graphs present mean ± SE. * *p* < 0.05, Student *t*-test. ns = non-significant.

Expanded bone-marrow-derived MSC were cultured in vitro for 7 days in serum free medium supplemented with or without 20 nM PACAP (1–27). A one-week incubation of MSC with PACAP did not change the expression of cell surface MSC markers: PACAP-treated MSC expressed typical markers as we previously reported for naïve MSC [16]. In flow cytometry analysis murine MSC were found to be negative for CD45 (<8%) and CD11b (<5%) and positive for CD106 (>50%), CD29 (>80%), CD44 (>70%), CD73 (>60%) and sca-1 (>40%) (Figure 1A). Both naïve and PACAP-treated MSC expressed mRNA for vasoactive intestinal peptide -2 (VPAC2) but not for VPAC1 and PACAP receptor type-I (PAC1), the currently known receptors for PACAP (Figure 1B). Since polarization of MSC to MSC1 and MSC2 is achieved through TLR4 or TLR3 activation, respectively, we tested their relative gene expression in PACAP-treated MSC. PACAP treatment increased the gene expression ratio of TLR3 versus TLR4 compared to naïve non-treated MSC (Figure 1E).

**Figure 2:**
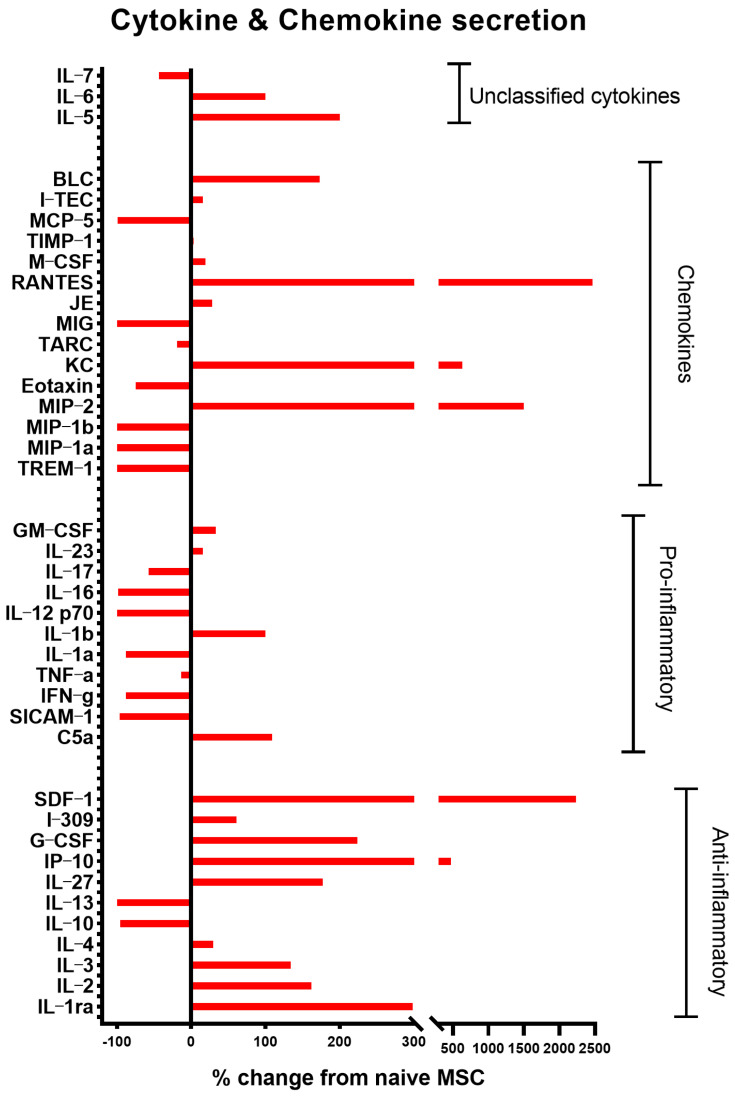
PACAP polarizes MSC towards an anti-inflammatory (MSC2) phenotype. Conditioned medium from naïve and PACAP-treated MSC was analyzed for chemokines and cytokines secretion, utilizing Proteome Profiler Mouse Cytokine Array (R&D Systems). The following chemokine and anti-inflammatory cytokines IL-2, IL-3, IL-4, IL-27, IP10, IL-1ra, RANTES, SDF-1, CCL2(JE), CCL1 (i-309), G-CSF, BLC were upregulated, while pro-inflammatory cytokines were downregulated (IL-6, IL-1a, IFN-ɣ and soluble ICAM-1). Analysis was performed on pooled samples (n = 3) for each culture.

Conditioned medium harvested from the last 24 h of culture was assayed for cytokine secretion using the Proteome Profiler Mouse Cytokine Array Kit (R&D). As seen in Figure 2, the cytokine secretion profile differs between naïve and PACAP-treated MSC. In the PACAP-treated cells, the anti-inflammatory chemokines and cytokines IL-2, IL-3, IL-4, IL-27, IP10, IL-1ra, RANTES, SDF-1, CCL2(JE), CCL-1 (i-309), G-CSF and BLC were over-expressed, while the pro-inflammatory cytokines IL-17, IL-1a, IFN-ɣ and soluble ICAM-1 were downregulated (Figure 2). Among these cytokines, the increased expression of IP10 (CXCL10), RANTES, IL-1ra and IL-4 was previously demonstrated for MSC2 with an immunosuppressive phenotype [15]. Thus, PACAP-treated MSC display clear MSC2-like features.

### 2.2. Systemic Administration of Polarized MSC Reduced the Levels of Pro-Aging Chemokines in the Serum of Aged Mice

**Figure 3:**
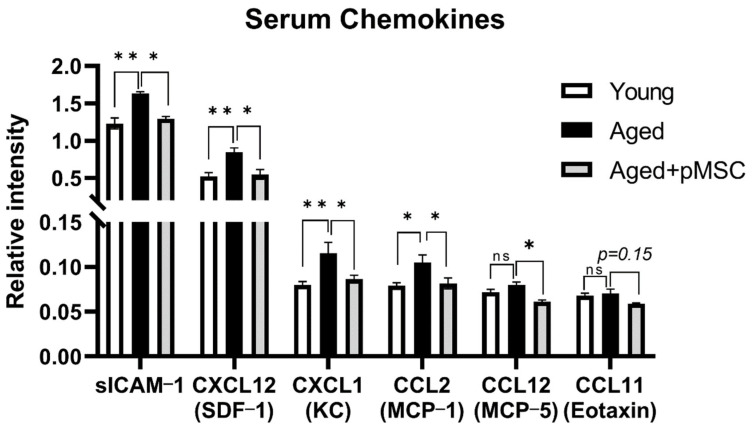
Systemic administration of polarized MSC to aged mice normalizes systemic chemokine levels. Intravenous injection of polarized anti-inflammatory MSC (pMSC, N = 3–4) reduces the levels of plasma chemokines that are associated with aging and inflammation [4] to levels of young (3 months old) mice (N = 7–8) compared with vehicle-treated aged control mice (N = 3), as detected by Proteome Profiler Mouse Cytokine Array (R&D Systems). Bars in the graph present mean ± SE. * *p* < 0.05, ** *p* < 0.01. One-way ANOVA. ns = non-significant.

To assess whether polarized MSC indeed affect the systemic immunological environment associated with brain aging, polarized (PACAP-treated) MSC were injected (2 × 10^5^ cells) into the tail vein of aged (18-months old) ICR mice. Three weeks later, serum cytokine levels were examined using the Proteome Profiler Mouse Cytokine Array kit. We noticed that polarized MSCs reduced the serum levels of the following chemokines: sICAM-1, CXCL12(SDF-1), CXCL1(KC), CCL2(MCP-1) all are known to be upregulated during aging. This reduction normalized plasma levels to that of normal young (3 months old) animals (Figure 3). Moreover, CCL12(MCP-5), a pro-aging chemokine [4], though not being increased in 18-month-old mice, was nevertheless reduced by the polarized MSC treatment (Figure 3). Similarly, CCL11(Eotaxin), another known pro-aging chemokine (Smith LK et al., 2017), showed a trend towards reduced levels following polarized MSC treatment (Figure 3).

### 2.3. Systemic Administration of Polarized MSC Increased Hippocampal Neurogenesis in Aged Mice

**Figure 4:**
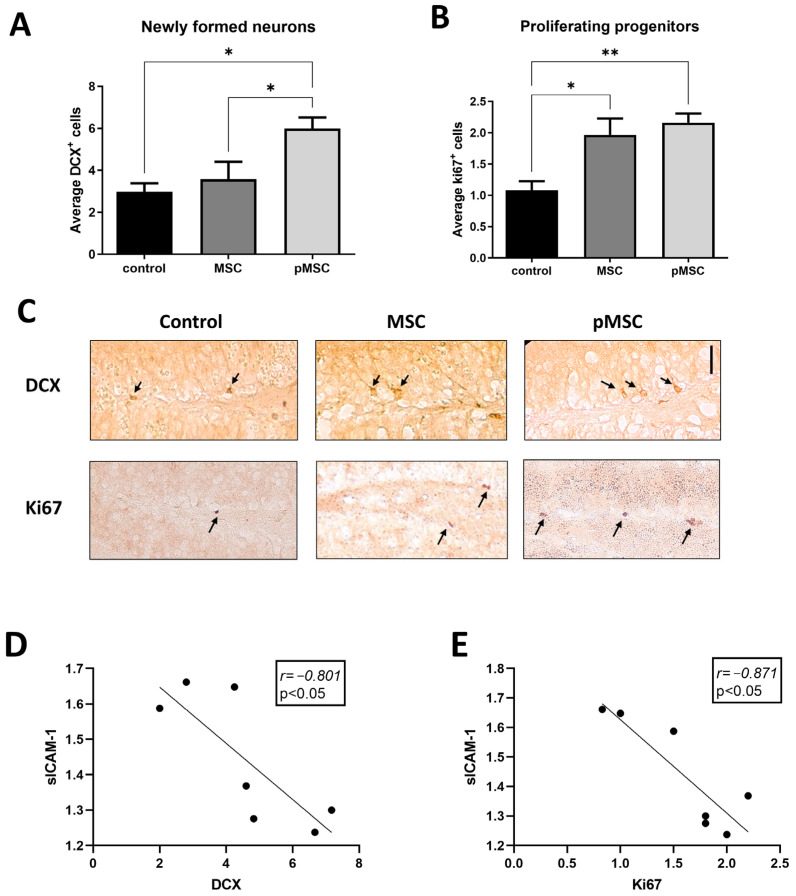
Systemic administration of polarized MSC increases neurogenesis in aged mice. To assess the therapeutic potential of anti-inflammatory MSC, we administered 2 × 10^5^ naïve (MSC) and polarized PACAP-treated MSC (pMSC) intravenously to 18-months aged mice. Immunohistochemistry for newly formed neurons expressing doublecortin in the granular cell layer was significantly increased in pMSC-treated animals (N = 5) compared with naïve-MSC- (N = 5) and vehicle-treated aged mice (N = 5) (**A**). Immunohistochemistry for proliferating neuro-progenitors (Ki67^+^) in the sub-granular zone of the dentate gyrus demonstrated increased number of cells in the hippocampus of mice treated with either naïve (N = 4) or polarized MSC (N = 6) compared with vehicle-treated aged mice (N = 4) (**B**). (**C**). Representing micrographs of DCX^+^ and Ki67^+^ cells in the dentate gyrus of the different treatment groups. Scale bar 20 μm. Arrows indicate positive cells and nuclei.. Linear regression graphs depicting the correlation between DCX^+^ and Ki67^+^ cell numbers and sICAM levels (**D**,**E**), respectively. Bars in graphs represent mean ± SE. * *p* < 0.05, ** *p* < 0.01, one-way ANOVA. Correlation was calculated using Pearson test.

We further assessed whether polarized MSC influence the reduced hippocampal neurogenesis, one of the characteristics of brain aging. Three weeks following the administration of polarized MSC, but not of naïve MSC (2 × 10^5^ cells i.v.), increased hippocampal neurogenesis was evident, as reflected by an increase in the number of DCX^+^ newly formed neurons in the granular cell layer of the dentate gyrus (Figure 4A). Additional staining for proliferating Ki67^+^ progenitors in the subgranular zone showed an increase in proliferating cells in aged male mice treated with both polarized MSC and naïve MSC (Figure 4B). Interestingly, inverse correlation was observed between sICAM-1 serum levels and the number of DCX^+^ cells (*Pearson r* = −0.8, *p* < 0.05, *n* = 7) and Ki67^+^ cells (*Pearson r* = −0.87, *p* < 0.05, *n* = 7) in the dentate gyrus of aged animals (Figure 4C,D).

### 2.4. Systemic Administration of Polarized MSC Improves Cognitive Function in Aged Mice

**Figure 5:**
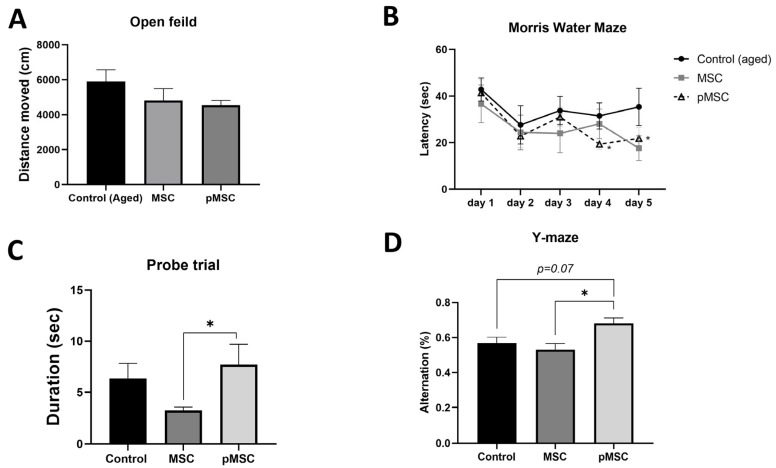
Systemic administration of polarized MSC improves cognitive function in aged mice. To assess the therapeutic potential of anti-inflammatory MSC, we administered 2 * 10^5^ naïve (MSC) and polarized PACAP-treated MSC (pMSC) intravenously to 18-months aged mice. No significant differences were observed between the different groups in general locomotion activity, as assessed in the open field test (**A**). Morris water maze assay demonstrated significant improvement in locating the hidden platform in pMSC-treated animals at days 4 and 5 compared with day 1 (**B**). The probe trial assay following the 5-day Morris water maze demonstrated significant increased localization in the platform zone in pMSC-treated animals compared with naïve-MSC-treated animals (**C**). Administration of pMSC also resulted in improved memory performance, as reflected by the increased alteration ratio in the Y-maze assay (**D**). Graphs present data as mean ± SE. N = 5 for all groups. * *p* < 0.05, one-way ANOVA. Correlation was calculated using Pearson test.

Finally, we tested the effect of polarized MSC systemic administration on behavior. Three days following the injection, mice were exposed to a series of behavioral assays. General locomotion activity assessed by the total distance traveled by the mice in the open field test was not found to differ between the different treatment groups (Figure 5A). Control non-treated aged mice displayed impaired spatial learning in the Morris water maze assay (Figure 5B). Over the 5-day assay, no statistically significant differences were detected in the latency durations obtained on days 2–5 compared with day 1 (repeated measures two-way ANOVA with Tukey’s multiple comparison test). However, in aged mice administered with polarized MSC, significant improvement was observed at days 4 and 5 of the assay (*p* < 0.05). A milder effect was noted in aged animals injected with naïve MSC, and while their improvement on day 5 was not significant compared with day 1, their scores on day 5 did not differ from the scores obtained by polarized MSC-treated animals. However, in the probe trial, MSC-treated animals displayed a significantly lower platform quadrant duration than animals treated with polarized MSC (Figure 5C).

**Figure 6:**
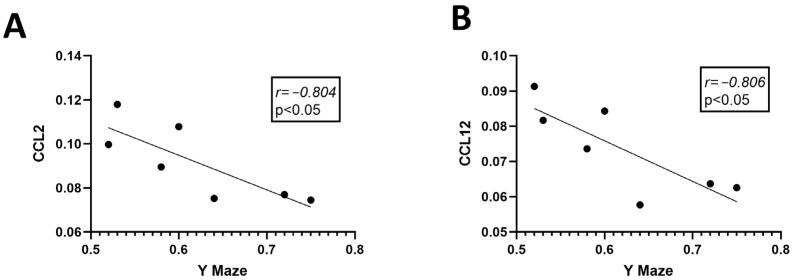
Cognitive behavior correlates with chemokine serum levels in aged mice. Linear regression graphs depicting the correlation between CCL2 and CCL12 serum levels and behavioral performance in the Y-maze test (**A**,**B**), respectively (N = 7). Correlations were calculated using Pearson test.

In the Y-maze paradigm, aged mice administered with polarized MSC demonstrated significantly better results, with an increased percentage of arm alterations, than did control aged or naïve-MSC-treated mice (Figure 5D). Notably, an inverse correlation was observed between aged mice performance in the Y-maze paradigm and plasma levels of the ‘pro-aging’ chemokines CCL2/MCP-1 (*Pearson r* = −0.806, *p* < 0.03, *n* = 7) and CCL12/MCP-5 (*Pearson r* = −0.804 *p* < 0.03, *n* = 7), corresponding with the putative influence of these plasma cytokines on hippocampal neurogenesis and cognitive functions (Figure 6).

### 2.5. Naïve and Polarized MSC Engraft to the Brain, Lungs and Liver following Intravenous Injection to Mice

**Figure 7:**
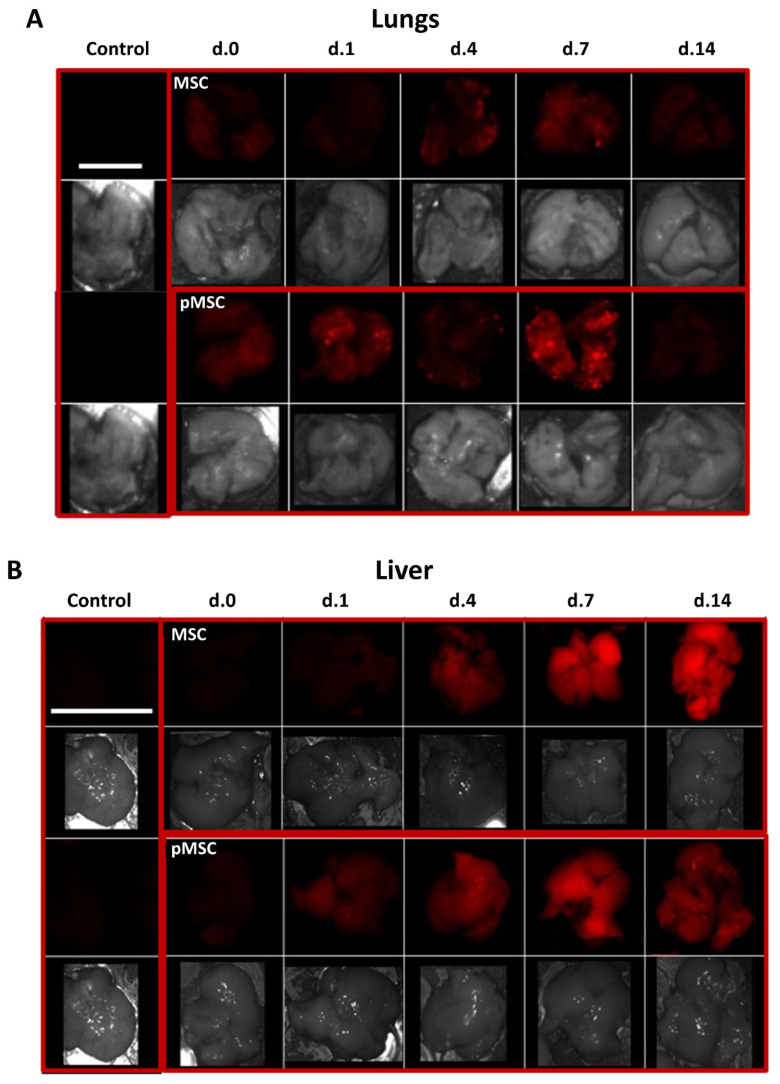

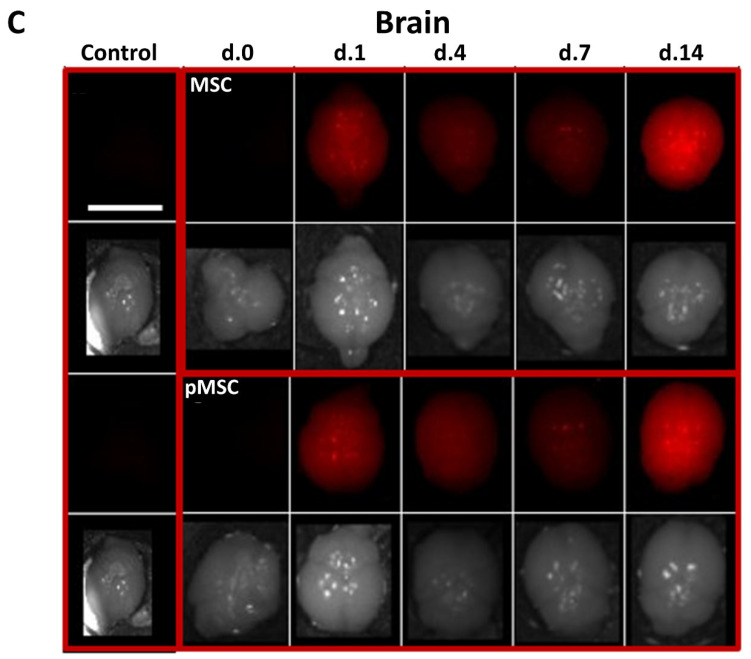
MSC engraft to various organs following intravenous injection in mice. DiR labeled naïve and polarized MSC (2 × 10^5^) were injected into the tail vein of 3-month-old mice. Labeled cells were detected using the Maestro in vivo imaging system at days 0, 1, 4, 7, and 14 following the injection. Imaging was performed and presented for the following dissected organs: (**A**) lungs (scale bar = 1 cm), (**B**) liver (scale bar = 3 cm), and (**C**) brain (scale bar = 1.5 cm). In each organ, the negative control (vehicle injected animal) is presented in the left rectangle, naïve MSC engrafted organ in the upper right and polarized MSC (pMSC) in the lower right rectangle. In each rectangle, the upper photographs represent fluorescent detection of DiR signal in the organ and the corresponding lower photograph, a visual light image of the same organ.

To determine the survival and distribution of naïve and polarized MSC following systemic administration, we injected 2 × 10^5^ DiR labeled naïve and polarized MSC into the tail vein of 3-month-old ICR mice. Labeled cells were tracked in various organs following dissection using the Maestro imaging system at days 0, 1, 4, 7 and 14 after injection. Labeled cells were tracked in the lungs 2 h following injection (day 0) and maintained through day 7 (Figure 7A). From day 1 through 14, cells were also traced in the liver and brain (Figure 7B,C). Overall engraftment to the lungs was more prominent in pMSC than naïve MSC, peaking at day 7, while engraftment to the liver was more prominent in naïve MSC than pMSC, peaking at day 14. Engraftment to the brain was prominent in both naïve and polarized MSC only by day 14 (Figure 7C).

## 3. Discussion

MSCs are well known for their immunomodulatory properties as they can induce both immune suppression and immune activation [11,13]. It was proposed that MSCs can be polarized to MSC1 (immune activation) and MSC2 (immune suppression) by activation of TLR4 and TLR3, respectively [14,15]. Pituitary adenylate cyclase-activating polypeptide (PACAP) is a neuropeptide with well-known anti-inflammatory properties [17]. Indeed, PACAP may also play a key role in the anti-inflammatory response induced by MSC [18]. It was further demonstrated in vitro that PACAP treatment can increase TLR3 gene expression in monocytes [19]. Similarly, we have found that short-term treatment of MSC with PACAP in vitro increased the TLR3/TLR4 gene expression ratio and polarized MSC towards a phenotype resembling MSC2, as indicated by the expression of anti-inflammatory cytokines (Figure 1 and Figure 2). PACAP-treated MSC retained their mesenchymal features, as observed in the similar marker expression in naïve MSC (Figure 1A). Interestingly, both naïve and polarized MSC expressed VPAC2 receptor for PACAP, suggesting its role in mediating the anti-inflammatory phenotype by PACAP. Indeed, it was previously shown that overexpression of VPAC2 in T cells promotes their polarization to Th2 with anti-inflammatory cytokine expression [20]. Previous reports have only found PAC1 to be expressed in human MSC following treatment with the pro-inflammatory cytokine INF-γ [18].

Age-related decline in cognitive function is associated with reduced hippocampal neurogenesis caused by changes in the systemic immune milieu [4,21]. These changes involve a decrease in plasma level of ‘pro-youth’ factors such as GDF-11 and an increase in ‘pro-aging’ factors, mainly inflammatory chemokines such as CCL11, CCL12, CCL19, CCL2, β2-microglobulin and haptoglobin. Other pro-inflammatory changes during aging also contribute to the effect of aging on the central nervous system and on hippocampal neurogenesis [3,22,23]. It is, therefore, reasonable to assume that anti-inflammatory strategies would be beneficial in promoting neurogenesis in aged animals. However, conventional treatment with anti-inflammatory drugs failed to elicit neurogenesis in a previous study [24]. On the other hand, targeting hippocampal neurogenesis using stem cells can be a promising approach. Indeed, we have previously shown in murine models of neurodevelopmental impairment that resemble premature aging with impaired neurogenesis that MSC administration to the CNS can restore neurogenesis [16,25]. Furthermore, local intracerebroventricular transplantation of MSC increased hippocampal neurogenesis and improved spatial learning in aging rats [26]. Park et al. (2013) demonstrated improved cognitive functions in aged mice intravenously administered with adipose-tissue-derived MSC, however, engraftment rates to the hippocampus were lower than intracerebroventricular administration and depended on multiple administrations [27]. The concerns were also raised regarding the effectiveness of intravenous administration and the engraftment of MSC in aged animals following stroke [28].

We, thus, tested the systemic administration of our polarized MSCs in aged mice and, indeed, found that polarized MSC treatment reduced the serum levels of three ‘pro-aging’ chemokines (CCL11, CCL12 and CCL2, Figure 3). In parallel, polarized MSC administration resulted in the elevation of hippocampal neurogenesis and improvement in cognitive functions (Figure 4 and Figure 5). In contrast, naïve MSC administration had a milder effect on neurogenesis and, similarly, had a milder effect on cognitive function as assessed by the behavioral assays. Although we did not analyze chemokine levels following naïve MSC administration and, therefore, cannot exclude their effect on systemic chemokine expression, the overall results imply that polarized MSC are more suitable than naïve MSC for treating brain aging.

Previously, similar results were obtained with umbilical cord blood MSC that were shown to increase neurogenesis and cognitive function following serial intraperitoneal injections in a rat model of aging [29]. The authors of this study attributed the effect to secreted factors, typical for ‘young’ MSC, as no long-term engraftment of the cells in body organs was observed. Indeed, systemic delivery of MSC results mainly in engraftment to the lungs and spleen with the therapeutic effect attributed mainly to their secretome [8,30]. As opposed to that, two studies performed on aged mice and D-galactose-induced brain aging in rats demonstrated engraftment and neural differentiation of intravenously administered MSC following multiple injections [27,31]. Fabian et al. (2017) demonstrated long-term engraftment of MSC to the cortex but not to the hippocampus of aged mice following a single intravenous injection, as detected by genomic PCR 28 days post injection. Furthermore, engraftment of aged MSC to the spleen and blood was also evident in aged mice [32]. In comparison to their observations, our study, conducted shortly following the injection, found significant engraftment of MSC to the CNS only by day 14 in 3-month-old mice. Engraftment to extra CNS organs, notably, the lungs and liver, was evident along the first two weeks following injection (Figure 7). It is, therefore, reasonable to assume that the changes in neurogenesis observed in our study arise from their influence on extra-hippocampal tissues, including blood, rather than on direct engraftment to the hippocampus.

In the present study, we suggest that, regardless of brain engraftment, polarized MSC can affect the systemic inflammatory milieu, which in turn, affect hippocampal neurogenesis and cognitive functions. The negative correlation found in the present study between sICAM-1, CCL2, CCL12 and newly formed DCX^+^ neurons, Ki67^+^ and Y-maze results (Figure 4 and Figure 6) suggest a mechanistic relation between plasma chemokines, hippocampal neurogenesis, and its related cognitive functions. We propose that by normalizing the levels of ‘pro-aging’ chemokines, polarized MSC can alleviate impaired neurogenesis and cognitive deficits in aged mice. Indeed, few studies demonstrated that blocking the expression of ‘pro-aging’ factors such as, β2-microglobulin and CCL2 in knockout mice resulted in increased neurogenesis [33,34]. Nevertheless, we do not exclude the possible effect of polarized MSC on neurogenesis and behavior by direct engraftment of the cells to the CNS.

We conclude that polarized anti-inflammatory MSC are better candidates for treating brain aging, via systemic administration, than naïve bone-marrow-derived MSC. Further studies should explore their therapeutic potential in other neurodegenerative and neuropsychiatric disorders associated with inflammation-related pathology.

## 4. Materials and Methods

### 4.1. Animals

All experimental procedures were performed in accordance with National Institutes of Health guidelines. MSC were isolated from 8 weeks old ICR mice. Behavioral and molecular experiments were conducted in 18-month-old male ICR mice. ICR mice were purchased from Harlan laboratories (Jerusalem, Israel). Food and water were provided ad libitum.

### 4.2. Mesenchymal Stem Cells Isolation and Culture

Mesenchymal stem cells (MSCs) were isolated from the bone marrow of 8-weeks old ICR mice. Briefly, tibias and femurs were removed and cleaned from connective tissue following animal euthanasia. Marrow was flushed out from epiphysis cut bones and disintegrated to cell suspension by passage through a series of needles (19 G, 21 G, 23 G and 25 G). Cells were then suspended in Dulbecco’s modified Eagle’s medium (DMEM) supplemented with 20% fetal bovine serum, 100 units/mL penicillin, 100 µg/mL streptomycin and 2 mM L-glutamine. Suspended marrow cells were plated in a 100-mm dish and cultured at 37 °C in 90% air + 10% CO_2_ atmosphere, with non-adherent cells removed 24 and 48 h following plating. For culture expansion, the same composition of medium was applied but with 10% FBS. Medium was changed twice weekly, cultures were subcultivated upon reaching confluence. MSCs were expanded in culture for up to 20 passages. Pituitary adenylate cyclase-activating peptide (PACAP) amino acids 1–27 was purchased from Bachem AG (Switzerland) cat. No. 127317-03-7. Polarization of MSC was achieved by treating MSC cultures with expansion medium supplemented with 20 nM of the neuropeptide PACAP (1–27) for 4 days. Cell culture reagents were purchased from Biological industries (Beit Haemek, Israel).

### 4.3. Flow Cytometry Immunophenotyping

To characterize the mesenchymal phenotype of polarized MSC, PACAP-treated MSC were immunophenotyped by FACS analysis (FACSCalibur with CellQuest software, Becton Dickinson, Franklin lakes, NJ, USA) using the mouse multipotent mesenchymal stromal cell marker antibody panel (cat No. SC018; R&D systems, Minneapolis, MN, USA), as we previously described [16]. Cultures were tested for the positive expression of the stromal markers CD29, CD44, CD73, CD106, SCA-1 and negative expression for the hematopoietic markers CD45 and CD11b.

### 4.4. Cytokine Array

A cytokine array detection assay was performed on cultured cell conditioned media or animal serum samples. Following 4 days of treatment with PACAP, cells were suspended in 5 mL serum-free DMEM and seeded into new 100 mm plates for an additional 24 h. The conditioned medium was collected, filtered through a 0.45 µm membrane and used for the analysis. Serum samples were prepared from blood samples collected directly from the heart following deep anesthesia of the animals induced by a mixture of ketamine (150 mg/kg) and xylazine (10 mg/kg). Serum was separated by centrifugation at 1000 RPM for 10 min. Serum samples were stored for long term at −20 °C. Cytokine expression was detected in 100 μL of serum or pooled conditioned media using the Proteome Profiler Mouse Cytokine Array Kit (R&D Systems, Cat no. SC018), according to the manufacturer’s protocol. Kit membranes were developed and imaged using the ImageQuant instrument. Images were analyzed using ImageJ software (Version 1.52a).

### 4.5. Behavioral Assays

For systemic administration of MSC, naïve and PACAP-treated MSC cultures were trypsinized and suspended in 0.9% saline. Then, 2 × 105 cells were injected into the tail vein of aged (18-months old) male mice. Three days following the injection, the mice were exposed to a series of behavioral assays to assess their behavior. Control animals were injected with vehicle only.

#### 4.5.1. Open Field Test

The general locomotor activity of the mice was evaluated using the open field paradigm. The open field arena consists of a 40 × 40 × 40 cm plastic box. Mice were placed at the center of the arena and were allowed to travel freely for a period of 6 min. Mice movements (total distance walked, time spent in different arena parts) were detected, recorded and analyzed using EthoVision Version 16 (Noldus; Wageningen, Netherlands), computerized video tracking system. Between each test, the arena was cleaned with 70% ethanol.

#### 4.5.2. Morris Water Maze test

The Morris water maze (MWM) test was used to assess spatial learning and memory. The MWM arena consisted of a 100 cm diameter, 40 cm height black plastic pool filled with water at 23 ± 2 °C to a height of 25 cm, with a hidden platform near the edge. The arena was marked with equal distance geometrical marks. The test was conducted over 5 consecutive days, with each mouse being placed at 3 different points for 1 min with a 60-min interval between trials. If the mouse reached the platform before 1 min, the time was recorded, and the mouse was allowed to stay on the platform for an additional 20 s. If the mouse failed to escape, it was manually placed on the platform for 20 s. The escape latency time was recorded using the EthoVision XT Version 16 tracking system software. On the fifth day, after the third trial (trial 15), a probe trial was performed with the platform removed, and the mice were allowed to swim for 60 s. The total duration spent in the platform zone was measured and learning curves showing the decrease in latency as the days progressed were compared between groups.

#### 4.5.3. Y-Maze

In the Y-maze test, the mouse was placed in the middle of the maze at the three-arm junction and left for 5 min. All his entrances to any arm were detected using EthoVision XT tracking system software. The spontaneous alteration behavior index was calculated and defined as the number of variable entries of the animal divided into the number of potential variable entrances.

### 4.6. Immunohistochemistry

Immunohistochemistry was performed to assess the effect of MSC administration on hippocampal neurogenesis. After the completion of behavioral assays, 2 weeks post MSC injection, mice were sacrificed through intracardial perfusion with PBS, followed by 4% paraformaldehyde (PFA). The brains were then removed, fixed overnight and equilibrated in 30% sucrose phosphate buffer. Brain tissue sections (20 μm) were cut using a MEV Slee Semi-Automatic Cryostat (SLEE medical GmbH, Nieder-Olm, Germany). Frontal sections of the hippocampus were stained for doublecortin (DCX), a neuronal differentiation marker, and Ki-67, a proliferation marker, using an immunohistochemistry kit (Cat. No. ab2253; Millipore, Burlington, Ma, USA) according to the manufacturer’s protocol. The sections were refixed with 4% PFA for 10 min and treated with 3% H_2_O_2_ for 10 min to block endogenous peroxidase. They were then permeabilized with 0.01% Triton X-100 for 5 min, followed by a 45-min incubation with blocking solution (provided by the kit). The sections were then incubated overnight with primary rabbit polyclonal anti-DCX (Abcam, UK. Cat No. ab18723) diluted 1:1000 in 0.5% bovine serum albumin or primary rabbit polyclonal anti-Ki-67 (Abcam, UK. Cat No. ab15580) diluted 1:1000 in 0.5% bovine serum albumin at 4 °C. Subsequently, the sections were treated with a horseradish peroxidase (HRP) one-step polymer conjugated secondary antibody for 30 min at room temperature. Visualization was achieved through a 10-min incubation with 3,3′ Diamino-benzidine tetrahydrochloride (DAB) buffer and DAB chromogen. The sections were washed with PBS 3 times for 5 min between each step. Doublecortin positive cells in the granular cell layer of the dentate gyrus in the hippocampus were counted in six representative sections, and the average number of positive cells per dentate gyrus per section was calculated for each mouse. Ki67-positive cells in the subgranular zone of the dentate gyrus were counted in a similar manner. Micrographs were acquired using an OLYMPUS BX53 microscope (Tokyo, Japan) equipped with OLYMPUS camera U-TV0.5XC-3 with OLYMPUS CellSens imaging software (Version 1.18).

### 4.7. Real-Time PCR

Total RNA was extracted from MSC culture using an RNeasy Purification mini kit (QIAGEN, cat no. 74104) according to the manufacturer’s protocol. RNA was quantified according to absorbance at 260 nm, measured using the NanoDrop 2000. Then, 1 µg of total RNA was reverse transcribed using GoScript™ Reverse Transcription System (Promega, cat No. A5003). After reverse transcription, real-time PCR analysis by real-time PCR QuantStudio^TM^ machine was conducted for the expression of *TLR3* and *TLR4* using LightCycler SYBR Green I Master (Roche, cat No. 04887352001) according to the manufacturer’s instruction with 300 nM primers concentration. *GAPDH* gene was used for sample normalization. Amplification was performed under the following conditions: 5 s denaturation at 95 °C, followed by 30 s annealing at 59 °C and then followed by 30 s extension at 72 °C for a total of 40 cycles. Primer pairs used were *TLR3* forward 5′-TTGTCTTCTGCACGAACCTG-3′ and *TLR3* reverse 5′-CGCAACGCAAGGATTTTATT-3′; *TLR4* forward 5′-ACCTGGCTGGTTTACACGTC-3′ and *TLR4* reverse 5′-CTGCCAGAGACATTGCAGAA-3′; *GAPDH* forward 5′-GGGGCTCTCTGCTCCTCCCTGT-3′ and *GAPDH* reverse 5′-TGACCCTTTTGGCCCCACCCT-3′; *VPAC1* forward 5′-CAAGGATATGGCCCTCTTCA–3′ and *VPAC1* reverse 5′- TGATGAACACACTGGGCACT-3′; *VPAC2* forward 5′-CAGATGTTGGTGGCAATGAC-3′ and *VPAC2* reverse 5′-CCTGGAAGGAACCAACACAT-3′; *PAC1* forward 5′-GACCTGATGGGCCTAAATGA -3′ and *PAC1* reverse 5′-GCCAGAATCCCCTATGGTTT–3′. primers were synthesized commercially (Sigma-Aldrich). PCR product specificity was confirmed using melting curve analysis and relative gene expression was calculated as 2^(−ΔΔCT)^.

### 4.8. Cell Engraftment Analysis

Naïve and polarized (PACAP-treated MSC) were suspended and incubated with 1 mM DiR fluorescent dye (Applied Biosystems. Cat No. GC-C019) dissolved in PBS for 15 min at 37 °C followed by 5 min at 4 °C). Labeled cells were washed twice with PBS and resuspended in saline. Approximately 200,000 DiR labeled MSC were intravenously injected to the tail vein of 3-months-old ICR mice. Control animals were injected with vehicle only. At days 0 (2 h), 1, 4, 7 and 14 following injections, mice were sacrificed, dissected, and immediately imaged using the Maestro In Vivo Imaging System (CRi Maestro ll), DiR signal was visualized using 780 nm/810 nm ex/em filter.

### 4.9. Statistics

All data in graphs are presented as mean with bars representing standard error. Statistical significance between two groups was assessed using Student’s *t*-test. For multiple group analyses, one-way analysis of variance (ANOVA) followed by Tukey’s post-hoc test was performed. For Morris water maze tests, repeated measures one-way ANOVA was performed. Correlations were calculated using the Pearson correlation test. All statistical analysis were performed using Prism GraphPad software (Version 9.5.1).

## Data Availability

Not applicable.
